# Layer-Averaged Water Temperature Sensing in a Lake by Acoustic Tomography with a Focus on the Inversion Stratification Mechanism

**DOI:** 10.3390/s21227448

**Published:** 2021-11-09

**Authors:** Shijie Xu, Zhao Xue, Xinyi Xie, Haocai Huang, Guangming Li

**Affiliations:** 1 Hainan Institute, Zhejiang University, Sanya 572025, China; 22034053@zju.edu.cn; 2 Ocean College, Zhejiang University, Zhoushan 316021, China; xinyixie@zju.edu.cn; 3 Development Center, Qingdao Nation Laboratory for Marine Science and Technology, Qingdao 266237, China; zxue@qnlm.ac; 4 Pilot Qingdao National Laboratory for Marine Science and Technology, Qingdao 266061, China; 5 National Innovation Institute of Defense Technology, Fengtai District, Beijing 100071, China

**Keywords:** coastal acoustic tomography, vertical slice inversion, multi-path arrival identification, stratification mechanism, water temperature observation, inversion error analysis

## Abstract

Continuous sensing of water parameters is of great importance to fluid dynamic progress study in oceans, coastal areas and inland waters. The acoustic tomography technique can perform water temperature field measurements horizontally and vertically using sound wave travel information. The layer-averaged water temperature can also be measured with the acoustic tomography method. However, investigations focusing on the stratified mechanism, which consists of stratification form and its influence on inversion error, are seldom performed. In this study, an acoustic tomography experiment was carried out in a reservoir along two vertical slices to observe the layer-averaged water temperature. Specifically, multi-path sound travel information is identified through ray tracing using high-precision topography data obtained via a ship-mounted ADCP during the experiment. Vertical slices between sound stations are divided into different layers to study layer division inversion methods in different preset types. The inversion method is used to calculate the average water temperature and inversion temperature error of every layer. Different layer methods are studied with a comparison of results. The layer division principle studied in this paper can be used for layer-averaged water temperature sensing with multi-path sound transmission information.

## 1. Introduction

Coastal acoustic tomography (CAT) is an innovative technology for monitoring water temperature variations in the coastal area. It is a development of ocean acoustic tomography (OAT) for application in shallow, coastal seas [1,2,3]. Small-scale fluid dynamic and water parameter observations are getting more attention [4,5]. The CAT system developed by the Hiroshima University Acoustic Tomography Group has been widely used for oceanographic observation since 1995 [6]. Plenty of experiments have been conducted to measure depth-averaged water temperature or map temperature distribution vertically and horizontally with the CAT system [7,8,9,10,11,12,13]. Syamsudin et al. obtained a five-layer-averaged water temperature and flow velocity of the vertical profile successfully in the Bali Strait using two acoustic systems. The temperature field results of the vertical profile obtained using the inversion method agree with the CTD measurements [14]. Syamsudin et al. also conducted a one-way sound wave transmission experiment with two acoustic positions. The experiment successfully identified the travel time of three acoustic ray paths, and the sound propagation section between two stations was divided into four layers [15]. The depth-averaged water temperature of four layers was calculated with the inversion method, and internal solitary waves in the Lombok Strait were observed successfully. Yu et al. conducted observation experiments at the Jiulong River Estuary and compared the inverted water temperature results with fixed-point temperature measurement results. The relative standard deviation (RSD) was about 6% [16]. In acoustic tomography research, the water parameter profiling in the vertical section is inverted by solving inverse problems. The layer-averaged method is regarded as more accurate [17,18,19,20] in water progress sensing. In recent years, temperature observations with CAT have been performed in small-scale waters and achieved relatively satisfactory results. It is found that the inversion results of each layer have obvious correlation as the quantity of identified rays is small. What is more, the layer depth division principle has a huge impact on inversion results, yet few studies focus on this problem.

The resolution and extraction of multiple arrival paths are the basis for the construction of vertical profiling. By comparing the arrival peaks and the acoustic ray simulation results, the travel time and ray path information of different acoustic rays can be obtained. The ideal multi-path recognition and extraction result is the acoustic ray that penetrates and covers the entire observation section. However, it is often difficult to obtain high-quality acoustic ray data in practice. Therefore, in certain situations, studying the quality of the results of different stratification methods can provide a choice for stratification selection.

In order to explore the influence of the layer depth division methods on the inversion results of the water temperature field, based on the three-station acoustic transmission experiment conducted at the Huangcai Reservoir in Changsha, Hunan, water temperature profile along vertical section is studied using acoustic travel information acquired between two CAT stations. The vertical section between two CAT stations is divided into three layers, and the influence of the layer width on the inversion error is analyzed in this study. The detailed experimental process and setting have been introduced by Huang et al. [21], and this paper focuses on research concerning the layer method mechanism.

The content of this paper is structured as follows: in Section 2, the inversion method of water temperature along a vertical section is presented, the experimental setting is also introduced in this section; in Section 3, the layer-averaged water temperature is calculated with different layer depth division methods; and the concluding remarks are given in Section 4.

## 2. Method and Experiment

In this section, the layer average water temperature of different methods along a vertical slice is calculated to study the layer method’s mechanism. The inversion process is discussed in detail. Then, the different layer settings and process of acoustic ray tracing are introduced. Next, the two-station sound reciprocal transmission experiment that was conducted in a reservoir is introduced. The experiment successfully acquired high-quality data, where three acoustic ray paths were identified after cross-correlation of received acoustic data with the transmitted acoustic signal. The multi-path arrivals were then used to reconstruct layer-averaged water temperature.

### 2.1. Inversion Method 

**Figure 1:**
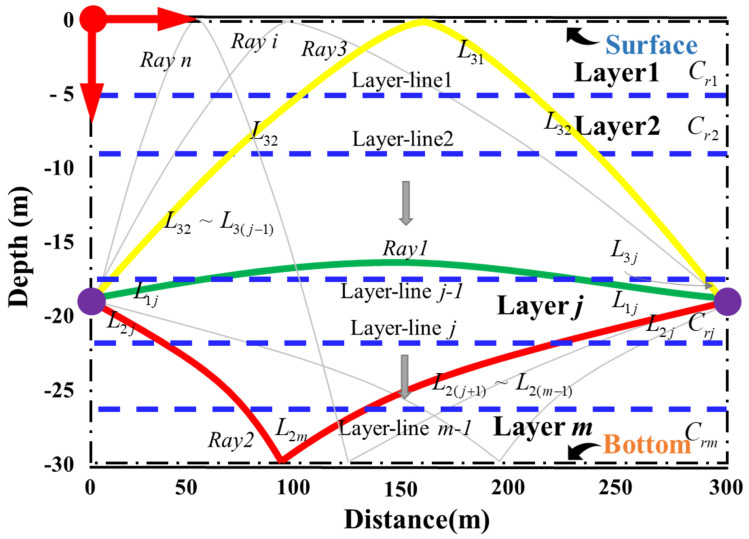
Reference ray simulation. $L_{ij}$ denotes the ray length of the *i*-th ray across the *j*-th layer. $C_{rj}$ denotes the reference acoustic speed of the *j*-th layer. The yellow line (Ray3) indicates a surface reflected ray. The green line (Ray1) indicates a direct ray. The red line (Ray3) indicates a bottom reflected ray. Gray lines are the sound ray that may exist but cannot be distinguished, which is not used for calculation. *m* denotes the total number of layers. *n* denotes the total number of rays.

The vertical slice was divided into three layers by two layer lines as seen in Figure 1. The bending direction of the acoustic ray was obtained from the TD data shown below. The research data in this paper are based on three identified rays between two stations, i.e., the direct ray (D), the bottom reflected ray (B), and the surface reflected ray (S), respectively. The water temperature in different layer can be reconstructed with inversion method [1,20,22].

For each ray, one can obtain:

(1) $$\frac{l_{1j}}{C_{rj}+\delta C_{j}}+\frac{l_{1(j-1)}}{C_{r(j-1)}+\delta C_{j-1}}+\cdot \cdot \cdot =t_{01}+\delta t_{1}\;\frac{l_{2j}}{C_{rj}+\delta C_{j}}+\frac{l_{2(j+1)}}{C_{r(j+1)}+\delta C_{j+1}}+\cdot \cdot \cdot +\frac{l_{2(m-1)}}{C_{r(m-1)}+\delta C_{m-1}}+\frac{l_{2m}}{C_{rm}+\delta C_{m}}=t_{02}+\delta t_{2}\;\frac{l_{31}}{C_{r1}+\delta C_{1}}+\frac{l_{32}}{C_{r2}+\delta C_{2}}+\cdot \cdot \cdot +\frac{l_{3(j-1)}}{C_{r(j-1)}+\delta C_{j-1}}+\frac{l_{3j}}{C_{rj}+\delta C_{j}}=t_{03}+\delta t_{3}\;\ldots \;\cdot \cdot \cdot \frac{l_{i(j-1)}}{C_{r(j-1)}+\delta C_{j-1}}+\frac{l_{ij}}{C_{rj}+\delta C_{j}}+\frac{l_{i(j+1)}}{C_{r(j+1)}+\delta C_{j+1}}+\cdot \cdot \cdot =t_{0i}+\delta t_{i}\;\ldots \;\frac{l_{n1}}{C_{r1}+\delta C_{1}}+\cdot \cdot \cdot +\frac{l_{n(j-1)}}{C_{r(j-1)}+\delta C_{j-1}}+\frac{l_{nj}}{C_{rj}+\delta C_{j}}+\frac{l_{n(j+1)}}{C_{r(j+1)}+\delta C_{j+1}}+\cdot \cdot \cdot +\frac{l_{nm}}{C_{rm}+\delta C_{m}}=t_{0n}+\delta t_{n}$$

where $\delta C_{j}$ denotes the reference sound speed deviation of the *j*-th layer. $t_{0i}$ and $\delta t_{i}$ denote the reference travel time and travel time deviation of the *i*-th ray, respectively. Taking Taylor expansion of Equation (1) and neglecting the second and higher order, we deduce the following:

(2) $$-\left(\frac{l_{1j}}{C_{rj}{}^{2}}\delta C_{j}+\frac{l_{1(j-1)}}{C_{r(j-1)}{}^{2}}\delta C_{j-1}+\cdot \cdot \cdot \right)=\delta t_{1}\;-\left(\frac{l_{2j}}{C_{rj}{}^{2}}\delta C_{j}+\frac{l_{2(j+1)}}{C_{r(j+1)}{}^{2}}\delta C_{\left(j+1\right)}+\cdot \cdot \cdot +\frac{l_{2(m-1)}}{C_{r(m-1)}{}^{2}}\delta C_{\left(m-1\right)}+\frac{l_{2m}}{C_{rm}{}^{2}}\delta C_{m}\right)=\delta t_{2}\;-\left(\frac{l_{31}}{C_{r1}{}^{2}}\delta C_{1}+\frac{l_{32}}{C_{r2}{}^{2}}\delta C_{2}+\cdot \cdot \cdot +\frac{l_{3(j-1)}}{C_{r(j-1)}{}^{2}}\delta C_{j-1}+\frac{l_{3j}}{C_{rj}{}^{2}}\delta C_{j}\right)=\delta t_{3}\;\ldots \;-\left(\cdot \cdot \cdot +\frac{l_{i(j-1)}}{C_{r(j-1)}{}^{2}}\delta C_{j-1}+\frac{l_{ij}}{C_{rj}{}^{2}}\delta C_{j}+\frac{l_{i(j+1)}}{C_{r(j+1)}{}^{2}}\delta C_{j+1}+\cdot \cdot \cdot \right)=\delta t_{i}\;\ldots \;-\left(\frac{l_{n1}}{C_{r1}{}^{2}}\delta C_{1}+\cdot \cdot \cdot +\frac{l_{n(j-1)}}{C_{r(j-1)}{}^{2}}\delta C_{j-1}+\frac{l_{nj}}{C_{rj}{}^{2}}\delta C_{j}+\frac{l_{n(j+1)}}{C_{r(j+1)}{}^{2}}\delta C_{j+1}+\cdot \cdot \cdot +\frac{l_{nm}}{C_{rm}{}^{2}}\delta C_{m}\right)=\delta t_{n}$$

Equation (2) can be rewritten as follows:

(3) $$\left[\begin{array}{c} \begin{array}{c} \delta t_{1}\\ \delta t_{2}\\ \delta t_{3} \end{array}\\ \ldots \\ \delta t_{i}\\ \ldots \\ \delta t_{n} \end{array}\right]=-[\begin{array}{ccccccccc} 0 & \ldots & \ldots & \frac{l_{1(j-1)}}{C_{r(j-1)}{}^{2}} & \frac{l_{1j}}{C_{rj}{}^{2}} & 0 & \ldots & 0 & 0\\ 0 & 0 & \ldots & 0 & \frac{l_{2j}}{C_{rj}{}^{2}} & \frac{l_{2(j+1)}}{C_{r(j+1)}{}^{2}} & \ldots & \frac{l_{2(m-1)}}{C_{r(m-1)}{}^{2}} & \frac{l_{2m}}{C_{rm}{}^{2}}\\ \frac{l_{31}}{C_{r1}{}^{2}} & \frac{l_{32}}{C_{r2}{}^{2}} & \ldots & \frac{l_{3(j-1)}}{C_{r(j-1)}{}^{2}} & \frac{l_{3j}}{C_{rj}{}^{2}} & 0 & \ldots & 0 & 0\\ & & & & \ldots & & & & \\ & & \ldots & \frac{l_{i(j-1)}}{C_{r(j-1)}{}^{2}} & \frac{l_{ij}}{C_{rj}{}^{2}} & \frac{l_{i(j+1)}}{C_{r(j+1)}{}^{2}} & \ldots & & \\ & & & & \ldots & & & & \\ \frac{l_{n1}}{C_{r1}{}^{2}} & \ldots & \ldots & \frac{l_{n(j-1)}}{C_{r(j-1)}{}^{2}} & \frac{l_{nj}}{C_{rj}{}^{2}} & \frac{l_{n(j+1)}}{C_{r(j+1)}{}^{2}} & \ldots & \ldots & \frac{l_{mm}}{C_{rm}{}^{2}} \end{array}]\left[\begin{array}{c} \delta C_{1}\\ \delta C_{2}\\ \ldots \\ \delta C_{j-1}\\ \delta C_{j}\\ \delta C_{j+1}\\ \ldots \\ \delta C_{m-1}\\ \delta C_{m} \end{array}\right]$$

Equation (3) can be expressed as

(4) y=Ex+n

where **y** denotes the column vector of travel time deviation, **x** denotes the column vector of sound speed deviation, **n** denotes errors from collecting travel times, and **E** denotes the coefficient matrix. Note that the problem as stated in Equation (3) can be easily solved.

The expected solution $\hat{x}$ is determined to minimize the objective function.

(5) $$J={\left(y-Ex\right)}^{T}(y-Ex)+\lambda x^{T}H^{T}Hx$$

where λ is the Lagrange multiplier. **H** is the regularization matrix constructed from the second-order derivative operator $\frac{\partial^{2}x}{\partial z^{2}}$.

(6) $$H=\left[\begin{array}{ccccc} -2 & 1 & 0 & \cdot \cdot \cdot & 0\\ 1 & -2 & 1 & \cdot \cdot \cdot & 0\\ \vdots & \ddots & \ddots & \ddots & \vdots \\ 0 & \cdot \cdot \cdot & 1 & -2 & 1\\ 0 & \cdot \cdot \cdot & 0 & 1 & -2 \end{array}\right]$$

However, for most cases, the number of layers and the number of rays do not have the same value. Then, the corresponding equation will be an ill-posed problem. Regularized inversion is introduced to solve the problem [4]. The excepted solution $\hat{x}$ is expressed as

(7) $$\hat{x}={\left({Ε}^{T}E+\lambda H^{T}H\right)}^{-1}E^{T}y$$

where λ is determined to make the squared residual (defined as ${\left\|\hat{n}\right\|}^{2}={\left\|y-E\hat{x}\right\|}^{2}$) smaller than a preset value and will be updated during each sound transmission process to catch the dynamic environment. The preset value is calculated as

(8) $$t_{n}=\frac{C_{n}L}{C_{0}{}^{2}}$$

where $t_{n}$ denotes the preset value, $C_{n}$ denotes the expected sound speed error, and $C_{0}$ denotes the average reference sound speed of the region.

After inversion, the inversion error for each layer can be calculated. The solution uncertainty P is formulated as

(9) $$P={\left(E^{T}E+\lambda H^{T}H\right)}^{-1}E^{T}\langle nn^{T}\rangle E{\left(E^{T}E+\lambda H^{T}H\right)}^{-1}$$

Then, the inversion errors $C_{err}$ for each layer can be calculated using

(10) $$C_{err}=diag(\sqrt{P})$$

The inversion error is an important factor to measure the accuracy and reliability of the inversion process. If the inversion error and the preset value are both satisfactory, the inversion problem is solved. Otherwise, the inversion process should be modified. Sometimes, there is no satisfied λ that can make the squared residual lower than the preset value, which indicates large errors of the inversion process.

In Section 3, the factors that affect the inversion process will be discussed with the data acquired from the two-station sound reciprocal transmission experiment. Special attention is paid to the layer division.

### 2.2. Experimental Settings

**Figure 2:**
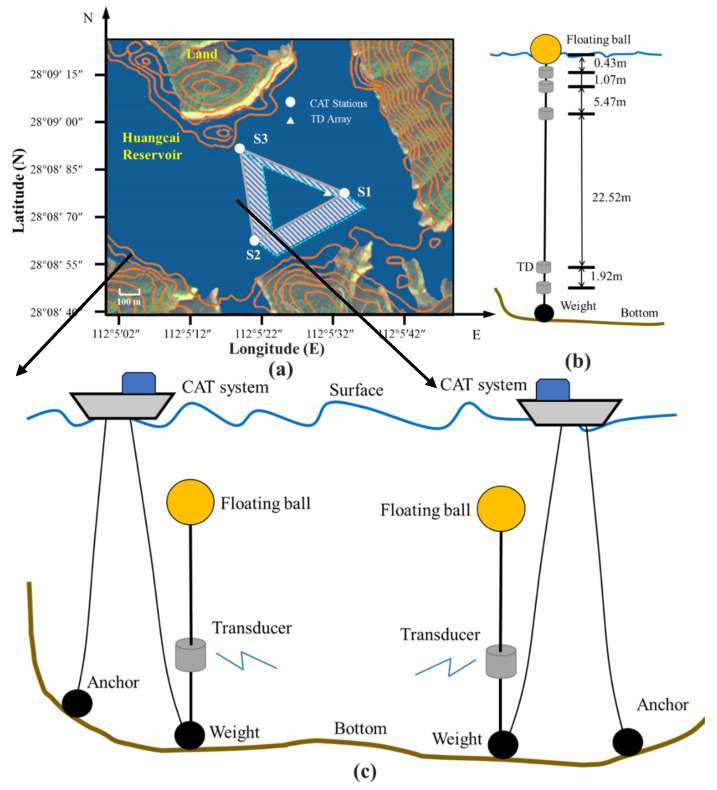
Experimental settings. (**a**) Experiment location and the layout of each station. The contour terrain in the figure is from 2015 data and the satellite map is from 2019 data, so they do not overlap completely. (**b**) The mooring mode of TD array. (**c**) The special mooring mode of CAT stations **S1** and **S2**.

A three-stations experiment was carried out in Huangcai Reservoir in Changsha, China, during 15–16 September 2020, and experimental setting is shown in Figure 2a. Three CAT stations with a distance of 270 m (**S1**–**S2**), 224 m (**S2**–**S3**), and 283 m (**S1**–**S3**) were deployed in the reservoir to transmit and receive acoustic signals. A temperature and pressure sensor (TD) array, as shown in Figure 2b, was deployed in the experimental area for water temperature sensing of different water depths. This study analyzes the layer-averaged water temperature between stations pair of **S1** to **S2**, **S2** to **S3**, and **S1** to **S3**, respectively. Transceivers of the CAT system were deployed at a depth of 16.9 m (**S3**) and 20 m (**S1**, **S2**), respectively. The special mooring mode is shown in Figure 2c. Every station was equipped with a floating ball and an additional weight to fix and maintain stability. This deployment method makes sure that the position of each transceiver of the CAT system remains constant when the boats move irregularly. Accordingly, the maximum drift distance of each transceiver is within 10 cm, which meets the requirement of high-precision inversion.

Previous investigation found that the bottom of the reservoir had a thick sedimentary layer. The thick sedimentary layer may increase the acoustic energy attenuation when acoustic waves interact with the bottom. Considering the presence of bottom reflection rays, a 10-order **M** sequence was selected to improve the signal-to-noise ratio (SNR) and meet simultaneous transmission requirements between short distances. The remaining parameters of this experiment are shown in Table 1. During the experiment, a TDS (temperature depth sensor) was used to measure the temperature profiling (the triangle in Figure 2), and shipborne ADCP (acoustic Doppler current profiler) was used to construct the experiment area terrain topography (the red arrow in Figure 2). Based on the results of direct acoustic path travel time and temperature profile [23], the distance between the two stations was calculated. More details and settings have been provided in [21].

This paper mainly studies different layer division types for calculating the average temperature of the layer along a vertical slice. The results between different stations and the types are studied, and the error and accuracy are also discussed in detail. The **S1–S2** and **S2–S3** stations with smaller topographical undulations were selected to analyze and compare the accuracy of the results.

**S1–S2** and **S2–S3** were divided into 5 types of 2 layers, 10 types of 3 layers, and 5 types of 5 layers. Table 2 shows five types of two-layer division. Table 3 shows 10 types of 3-layer division. Table 4 shows five types of five-layer division. The “Length of xx layer” in the table refers to the distance between different layer lines. For example, “Length of 1st layer” indicates the length from the surface to the first layer line. In the rest of the paper, data analysis is realized by processing each type.

### 2.3. Ray Simulation

**Figure 3:**
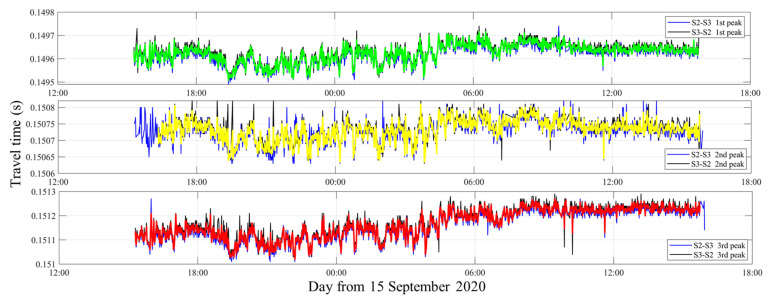
The travel time of three arrival peaks between **S2** and **S3**.

**Figure 4:**
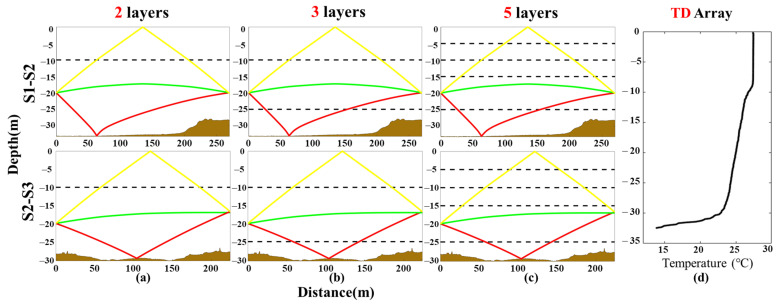
Ray simulations of different layer types in **S1–S2** and **S2–S3**. (**a**) Ray simulation of Number 2-2. (**b**) Ray simulation of Number 3-7. (**c**) Ray simulation of Number 5-2. (**d**) Temperature profiling.

Take **S2–S3** for example, the travel time of three arrival peaks between **S2** and **S3** was identified and extracted with correlation results, as shown in Figure 3. The green, yellow, and red curves indicate the travel time of the 1st, 2nd, and 3rd arrival peaks, respectively, corresponding to the direct rays (D), surface reflected rays (S), and bottom reflected rays (B). D, S, and B correspond to the green, yellow, and red acoustic ray paths in Figure 4, respectively.

Three typical ray simulation comparisons in **S1–S2** and **S2–S3** are shown in Figure 4. The temperature profiling (d) measured by the TD array shows that temperature suddenly changes by about 10 °C as the depth increases, i.e., closer to the bottom. Therefore, the maximum depth of the layer line was set at 25 m. The main difference between different layer methods is the acoustic ray simulation process. The multi-path resolution results in different layers are same, but the final inversion result will have a big difference.

Table 5 shows that the layer length and reference travel time of each ray in the two layers, three layers, and five layers of **S2**–**S3** correspond to Figure 3 and Figure 4, respectively.

### 2.4. Multi-Peak Identification

**Figure 5:**
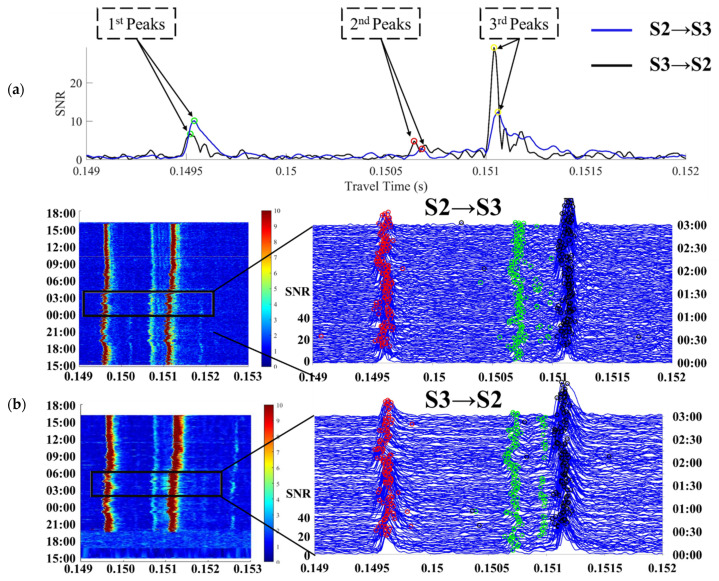
Multi-peak identification. (**a**) A set of cross-correlation results. (**b**)The special mooring mode of the CAT station.

In the process of multi-front identification [21], after two stations are correlated, the peaks with higher SNR were distinguished and identified. The cross-correlation results and multi-peak identifications of **S2**–**S3** during the experiment are stacked in Figure 5.

Figure 5a shows the cross-correlation result of a set of data. In Figure 5b, the left side show the colormaps of top view data, and the magnified figures on the right side are the overviews of stacked cross-correlation data. The green, yellow, and red circles dotted the peaks of direct path, surface reflected path, and bottom reflected path, respectively (from 0–3 o’clock on 16 September).

## 3. Results and Discussion

As discussed in Section 2, the comparison of average temperature curves and inversion errors under different layer types is the key point. In theory, the result quality of each layer is determined by the quality of acoustic ray path information. In this article, there are three steps for study and comparison, which are summarized as follows:

Step 1: Calculate temperature inversion with the preset value of temperature error less than 0.8 and less than 0.05 for all types, respectively. Compare the results under different layer types and eliminate the larger error types. Note that, in the remaining part of the paper the preset value of temperature error is abbreviated pvtem-er.

Step 2: Extract the inversion results and set comparation groups as follows: (1) The relationship between same layers and pvtem-er but different layer types. (2) The relationship between same layers and types but different pvtem-er. (3) The relationship between same pvtem-er and closer types but different layers.

Step 3: Summarize the results and analyze the rules in step 2. Explore more general experience.

### 3.1. Layer-Averaged Water Temperature of **S2–S3**

In order to better instruct the results, **S2–S3** data analysis was selected. Display three sets of data: same pvtem-er but different layer type of (a) and (b), same layer type but different pvtem-er of (b) and (c).

#### 3.1.1. Temperature Inversion Results of **S2–S3** Three Layers

**Figure 6:**
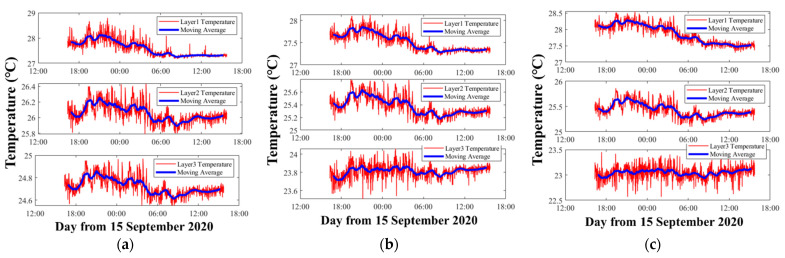
Average temperatures of three layers along a vertical slice. (**a**) Layer division results of Number 3-5. The pvtem-er wa smaller than 0.8 °C. (**b**) Layer division results of Number 3-7. The pvtem-er was smaller than 0.8 °C. (**c**) Layer division results of Number 3-7. The pvtem-er was smaller than 0.05 °C. The red curve indicates the layer average temperatures, the blue bold curve indicates 1 h moving average of the data.

The vertical average temperature inversion results with three kinds of representative three-layer divisions are mapped in Figure 6.

As shown in Figure 6, when the layer division and inversion settings are different, the results show large differences. Comparing Figure 6a with Figure 6b, the only difference is the layer division width of the bottom two layers. However, not only the bottom two layers show differences between the results, but the temperature results of the first layer also have big differences. The average temperature of the first layer in Figure 6a was 27.60 °C, while in Figure 6b it was 27.52 °C. Additionally, the trends of the third layer between Figure 6a and Figure 6b were quite different, which indicates particular fluctuations in the bottom.

Figure 6c satisfies stricter requirement during the inversion, which means that the λ was chosen to make the temperature error smaller than 0.05 °C during the inversion process. Consequently, compared with Figure 6b, the results of Figure 6c are assumed to be more precise.

Note that any λ in the inversion process corresponding to Figure 6a cannot satisfy the temperature error below 0.05 °C. Therefore, the layer division greatly affects the inversion process.

**Figure 7:**
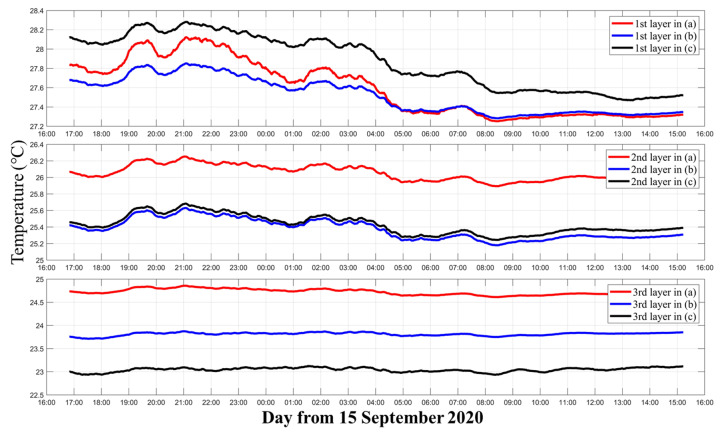
Moving average of the three layers’ temperature. The red, blue, and black curves indicate the three layers’ temperatures corresponding to Figure 6a–c, respectively.

The moving average temperature results of three layers corresponding to Figure 6a–c are shown in Figure 7. Figure 7 further illustrates the differences among the three different inversion settings. In the first layer, although they use the same data and calculate the temperature of the same layer, compared with Figure 6a and Figure 6b, the average temperature of Figure 6c was nearly 0.3 °C higher. In the second layer, Figure 6b,c contained most of the ray paths, so their results were close. On the contrary Figure 6a had less ray information, due to which the average temperature was nearly 0.8 °C higher, resulting in a large error. In the third layer, the average temperatures of Figure 6a–c were separated by nearly 1 °C.

**Figure 8:**
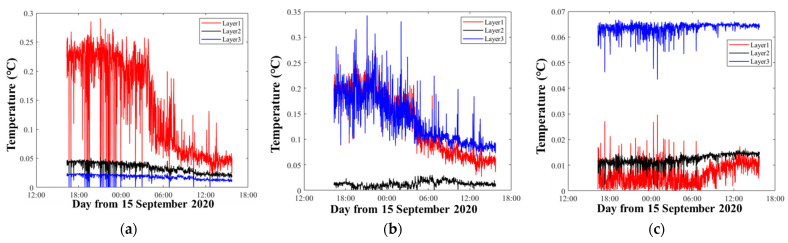
Temperature inversion errors. The red, black, and blue curves indicate the errors of the first layer, the second layer, and the third layer, respectively. (**a**–**c**) correspond to the results of Figure 6a–c, respectively.

Figure 8 shows the inversion errors that correspond to different layer divisions in Figure 6. The inversion errors were calculated from Equations (7) and (8). As expected, in the first layer, Figure 8a,b showed large errors during observations, while the errors of Figure 8c were small and stable. The other layers were as expected. Thus, the inversion errors were regarded to be reasonable to measure the quality of the inversion process, although different inversion settings were used.

#### 3.1.2. Temperature Inversion Results of **S2–S3** with Two Layers

**Figure 9:**
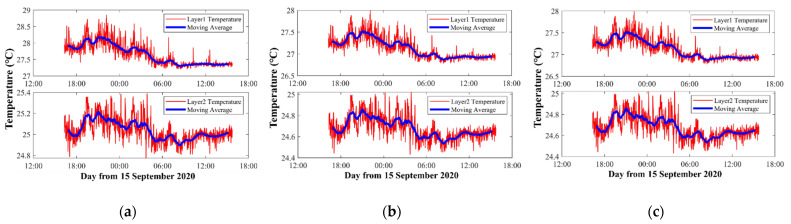
Two layers’ average temperatures along vertical slice. (**a**) Layer division results of Number 2-2. The pvtem-er was smaller than 0.8 °C. (**b**) Layer division results of Number 2-3. The pvtem-er was smaller than 0.8 °C. (**c**) Layer division results of Number 2-3. The pvtem-er was smaller than 0.05 °C.

The vertical average temperature inversion results with three kinds of representative two-layer divisions are mapped in Figure 9.

As shown in Figure 9, the result was similar to result presented in Section 3.1.1. Comparing Figure 9a with Figure 9b, the difference was the layer division width of the two layers. Therefore, the average temperatures of the first layer and second layer in Figure 9a were 27.66 and 25.04 °C, while in Figure 9b they were 27.13 and 24.68 °C. As for Figure 9b,c, the difference between their average temperature in the first and second layers was about 0.15 °C.

**Figure 10:**
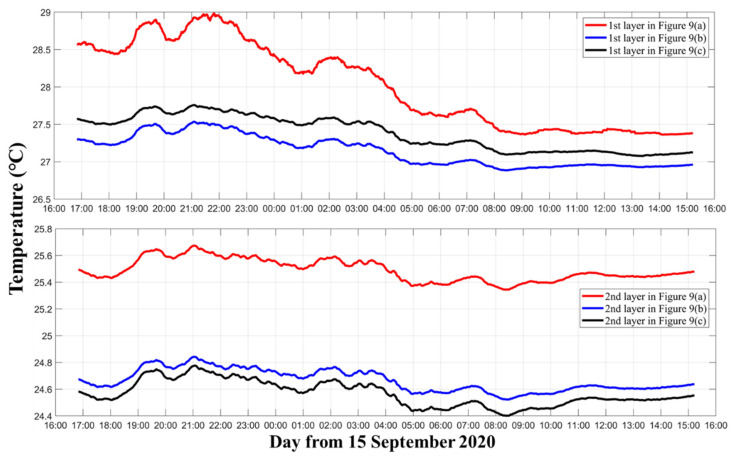
Moving average of the two layers’ temperature. The red, blue, and black curves indicate the two layers’ temperatures corresponding to Figure 9a–c, respectively.

The curve trend comparation between Figure 9a–c can be seen more clearly in Figure 10. Figure 10 shows the moving average temperature results of the two layers. In the first layer, the trends between Figure 9a with Figure 9b,c were quite different. In the second layer, the trends between Figure 9a–c are similar, but the temperature for Figure 9a is higher than that for Figure 9b,c. Comparing Figure 9b and Figure 9c, the curves had close trends, but the temperature at Figure 9b was higher in the first layer and that at Figure 9c was higher in the second layer.

**Figure 11:**
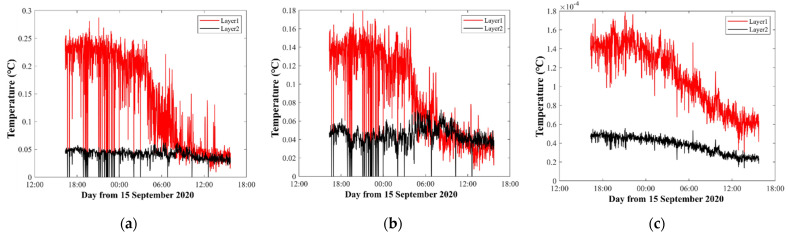
Temperature inversion errors. The red and black curves indicate the errors of the first layer and the second layer, respectively. (**a**–**c**) Correspond to the results of Figure 9a–c, respectively.

Figure 11 shows the inversion errors that correspond to different layer divisions in Figure 9. As above analyzed, in the first layer, Figure 11a,b showed large errors during observations, while the errors of Figure 8c were small. In the second layer, the error of Figure 11c was smaller than that of Figure 11a,b.

Theoretically, result in Figure 9c satisfied a stricter requirement during the inversion, which meant that the results were more accurate. However, the extremely small error of Figure 11c could not prove that the result of Figure 11c was reasonable. Therefore, further discussion is needed to establish whether the two-layer division is optimal.

#### 3.1.3. Temperature Inversion Results of **S2–S3** with Five Layers

**Figure 12:**
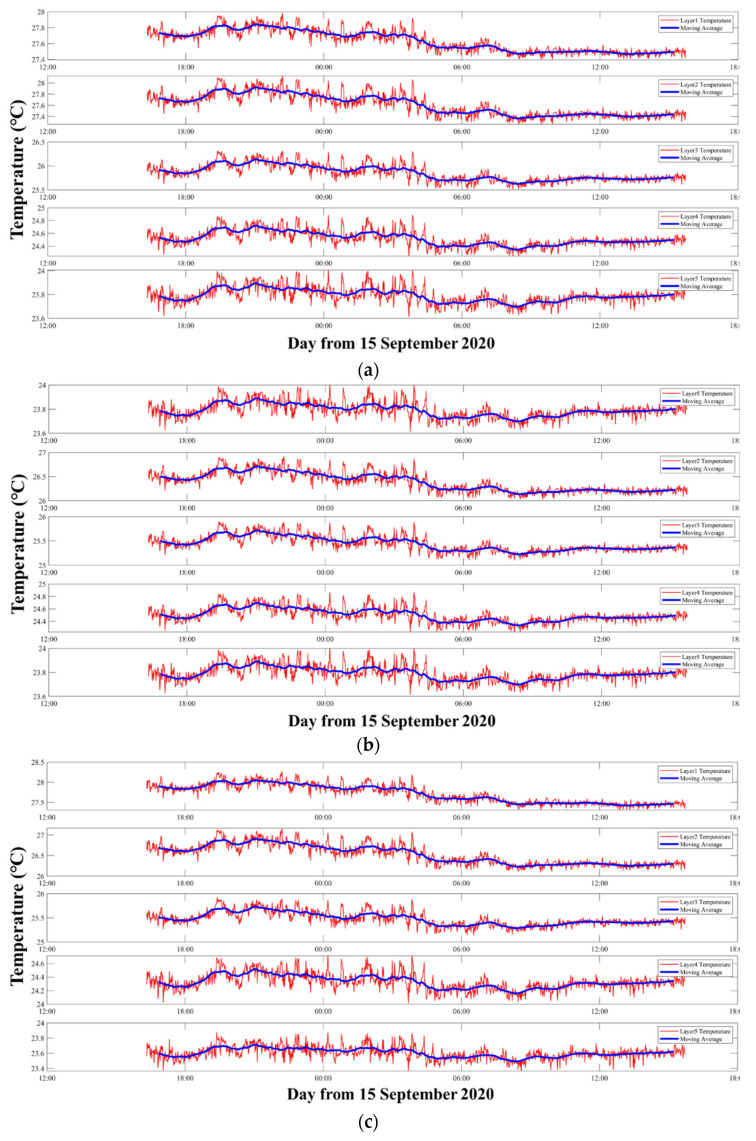
Five layers’ average temperatures along a vertical slice. (**a**) Layer division results of Number 5-3. The pvtem-er was samller than 0.8 °C. (**b**) Layer division results of Number 5-5. The pvtem-er was smaller than 0.8 °C. (**c**) Layer division results of Number 5-5. The pvtem-er was smaller than 0.05 °C.

The vertical average temperature inversion results with three kinds of representative five-layer divisions are mapped in Figure 12.

Figure 12 shows the five layers’ average temperatures along a vertical slice. Comparing with Section 3.1.1 and Section 3.1.2, the temperature curves in Figure 12a–c were more similar. Figure 12a,b have same layer division width, the average temperatures in Figure 12a and Figure 12b were 24.516 and 25.508 °C, respectively. However, the average temperatures in Figure 12a,b were 27.632 and 26.384 °C giving an error of about 1.3 °C in the first layer. As for Figure 12b,c, due to the same layer types, the average temperature in Figure 12c was 27.713 °C in the first layer. However, different pvtem-ers led to a 0.2 °C error in the fourth layer.

**Figure 13:**
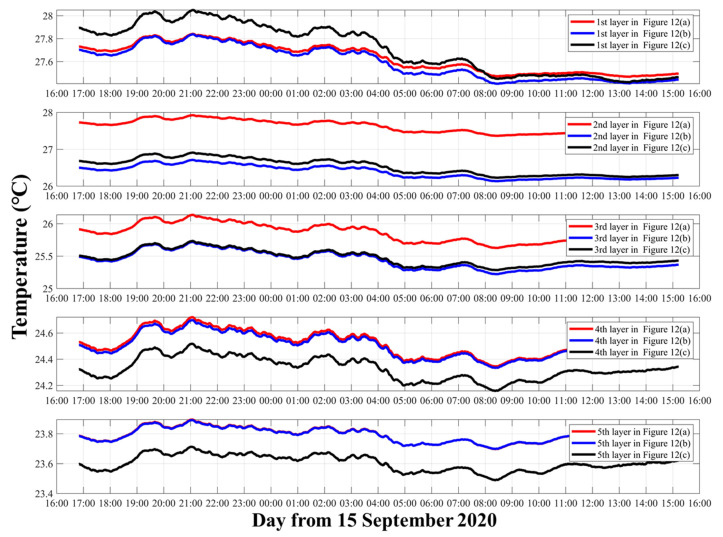
Moving average of five layers’ temperature. The red, blue, and black curves indicate five layers’ temperatures corresponding to Figure 11a–c, respectively.

From Figure 13, the curve trend of Figure 12a–c was more similar than that for Figure 7a–c and Figure 10a–c. Only the first layer in Figure 12a had trends that were different from those of Figure 12b,c. Although the curve trends were close, the error caused by the pvtem-ers still existed in every layer.

It can be concluded that, as the number of layers, increased the correlation of results increased in every layer. Therefore, in the five layers, although Figure 12c satisfied a stricter requirement during the inversion, it could not be considered as a good result. In particular, the trends of every layer were almost the same, showing that this setting was not an optimal solution for analysis.

**Figure 14:**
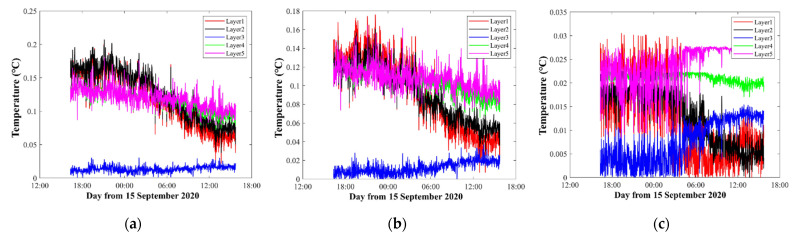
Temperature inversion errors. The red, black, blue, green and purple curves indicate the errors of the first layer, the second layer, the third layer, the fourth layer, and the fifth layer, respectively. (**a**–**c**) correspond to the results of Figure 12a–c, respectively.

Figure 14 shows the inversion errors that correspond to different layer divisions in Figure 12. Comparing Figure 14a–c, the temperature error of Figure 14a in the first layer was higher than that in Figure 14b,c. Additionally, the third layer had the smallest error in five layers. The errors almost showed a downward trend in all temperature inversion error figures, which led to speculation that the environment was relatively stable.

### 3.2. Comparison of **S2–S3**

As described in Section 3.1, 10 different layer divisions were calculated and compared. Based on the characters of inversion errors, we sorted the **S1**–**S2** and **S2**–**S3** results into two different groups.

Group 1 contained all layer divisions of two layers, three layers, and five layers.

Group 2 contained of the two layers: number 2-2, 2-3, and 2-4; of the three layers: number 3-3, 3-4, 3-6, 3-7, and 3-8; And of the five layers: number 5-2, 5-3, and 5-4.

The layer division numbers are shown in Table 2, Table 3 and Table 4 above.

**Figure 15:**
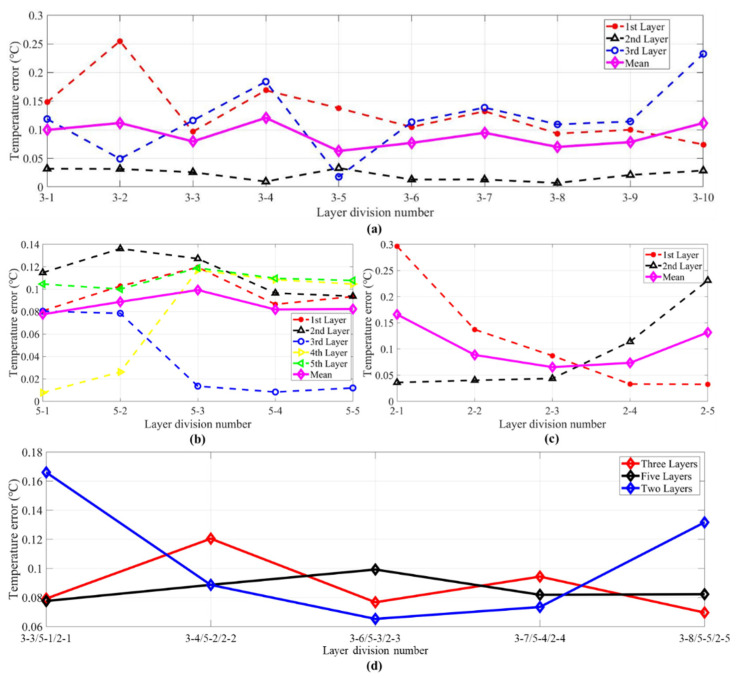
Inversion errors of the first group of **S2**–**S3**. (**a**) Temperature error of three layers. (**b**) Temperature error of five layers. (**c**) Temperature error of two layers. (**d**) Mean temperature error of three, five, and two layers. The red, black, blue, green, purple, and magenta curves in (**a**–**c**) indicate the mean errors of the first, second, third, fourth, fifth layer, and the mean errors of three layers, respectively. The red, black, and blue curves in (**d**) indicate three layers, five layers, and two layers.

The inversions of the first group were set to make the inversion errors smaller than 0.8 °C by controlling the preset value mentioned in Section 3.1. Figure 15a–d shows the temperature error results of every type. As can be seen in Figure 15a, the errors of the second layer were low for all conditions, while the first and the third layer fluctuated greatly. The maximum error of the first layer was for number 3-2. For this layer method, the first layer’s length was 5 m, while the others were 10 and 15 m long, respectively. The maximum error of the third layer was for number 3-10. For this kind of layer division, the third layer’s length was 5 m, which caused a large error fluctuation.

In Figure 8b, the fourth layer of 5-1 and 5-2, and the third layer of 5-3, 5-4, and 5-5 had smaller errors. The errors of the third layer and the fourth layer were opposite. The temperature error of the first, second, and fifth layers had less volatility. The average error in Figure 15b was close to the value in Figure 15a.

In Figure 8c, when there were only two layers, it can be seen that the error fluctuations between the groups were relatively large. Number 2-3 was divided equally two layers and had the smallest error. However, when the length of the two layers has a large difference (2-1 and 2-5), there will be a large error between the two layers.

Combining the figures above, the following conclusions can be obtained from Figure 15a–c: when the number of layers increases, the inversion error can be appropriately reduced. However, when the acoustic ray information is confirmed, too many layers will over-fit the results in layers, and the results will be deviated. Enlarged inversion errors may appear when the layer length is quite small, and the error is the smallest when the lengths of adjacent layers are close.

In Figure 15d, the conclusion above can be verified from the average error curves. The error fluctuation of two layers was obviously larger than that for the five layers and three layers. For more accurate evaluation, the selected types will be further discussed and verified in the second group.

**Figure 16:**
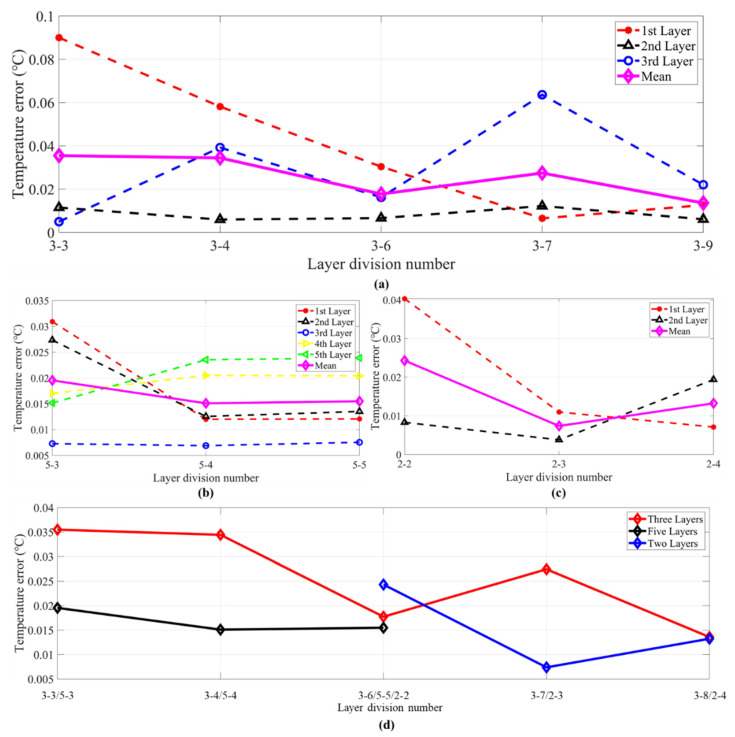
Inversion errors of the second group of **S2**–**S3**. (**a**) Temperature error of three layers. (**b**) Temperature error of five layers. (**c**) Temperature error of two layers. (**d**) Mean temperature error of three, five, and two layers. The red, black, blue, green, purple, and magenta curves in (**a**–**c**) indicate the mean errors of the first, second, third, fourth, fifth layer, and the mean errors of three layers, respectively. The red, black, and blue curves in (**d**) indicate three layers, five layers, and two layers.

However, the layer divisions of 3-1, 3-2, 3-5, 3-9 and 3-10, 2-1 and 2-5, 5-1 and 5-2 in Figure 15 could not make the inversion error as small as 0.05 °C during the inversion process. In contrast, the second group could all find appropriate λ to make the inversion error smaller than 0.05 °C. Figure 16 shows the inversion errors of the second group. Compared with Figure 15, the inversion errors are greatly decreased in Figure 16.

Comparing between two kinds of groups, the layer divisions of the second group in Figure 16a had one important characteristic: all layers contained at least one particular acoustic ray. Taking the layer division of Number 3-5 and Number 3-7 as an example, the first layers of the two divisions were the same. However, the second layer of Number 3-5 only had surface reflected ray, which was the same as the first layer. In other words, the first layer and the second layer both contained only one ray. In contrast, the length of the second layer for layer divisions in Number 3-7 was 15 m, which contained both a direct ray and a surface ray. For the layer division in Number 3-7, the particular acoustic rays were surface reflected ray, direct ray, and bottom reflected ray, which corresponded to the first layer, the second layer, and the third layer, respectively.

Comparing Figure 16b,c with the first group, the error of every layer was reduced. At the same time, it can be obtained from Figure 16d that the average error fluctuation was reduced. The error characteristics were similar to those in Figure 15. Consequently, the first layer division principle should be as follows: it is better for every layer to contain one particular acoustic ray, and two layers that contain only one, i.e., the same, acoustic ray should be avoided.

As shown in Figure 16, the errors of the second layer were also low compared with that in the others. As discussed in Section 2.1, the H matrix was used to smooth the solution through a moving average of three consecutive layers, which may explain the low inversion errors of the second layer.

Furthermore, it can be seen from the three-layer setting that the inversion errors of the first layer were high in the layer divisions of 3-3 and 3-4, and the errors of the third layer were high in 3-7 and 3-9. These high errors all corresponded to small layer length. Obviously, the inversion errors were also related to the ray length across each layer when the first principle was satisfied. In addition, the greater the corresponding reduction in the inversion error of every layer increases with layer numbers. 

**Figure 17:**
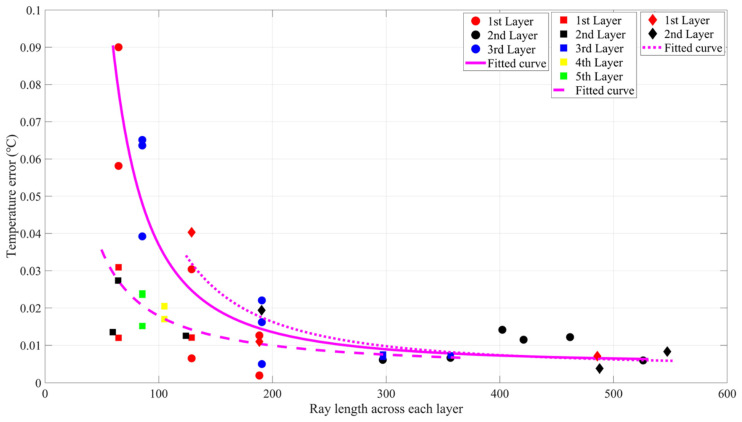
Relationship between the ray length across each layer and inversion errors of **S2**–**S3**. Three layers: Red, black, and blue circles denote the errors of the first layer, the second layer, and the third layer, respectively. The magenta curve indicates the fitted curve by using the power function. Five layers: Red, black, blue, yellow, and green squares denote the errors of the first layer, the second layer, the third layer, the fourth layer, and the fifth layer, respectively. The magenta dotted curve indicates the fitted curve by using the power function. Two layers: Red and black diamonds denote the errors of the first layer and the second layer, respectively. The magenta dotted curve indicates the fitted curve by using the power function.

In the second group, the relationships between the ray length across every layer and the inversion errors are displayed in Figure 17. The inversion errors decreased quickly when the length of rays across each layer increase. The data were fitted using the power function, which can be expressed as follows:

(11) $$\mathrm{Three}\;\mathrm{layers}:\;{\hat{T}}_{err}=202.8*L^{-1.901}+0.005\;\mathrm{Five}\;\mathrm{layers}:\;{\hat{T}}_{err}=2.787*L^{-1.141}+0.0035\;\mathrm{Two}\;\mathrm{layers}:\;{\hat{T}}_{err}=247.43*L^{-1.874}+0.0043$$

By Equation (11), the second principle to divide the vertical can be summarized: every layer should be divided to include roughly the same ray length to minimize the inversion errors. At the same time, the third principle can be inferred that inversion error decreases as the layer number increases.

Looking back at the results of Figure 15 and Figure 16, the minimum two errors were in the three-layer division of 3-6 and 3-8, whose ray lengths crossing the three layers are of same equality.

### 3.3. Comparison of **S1–S2**

**Figure 18:**
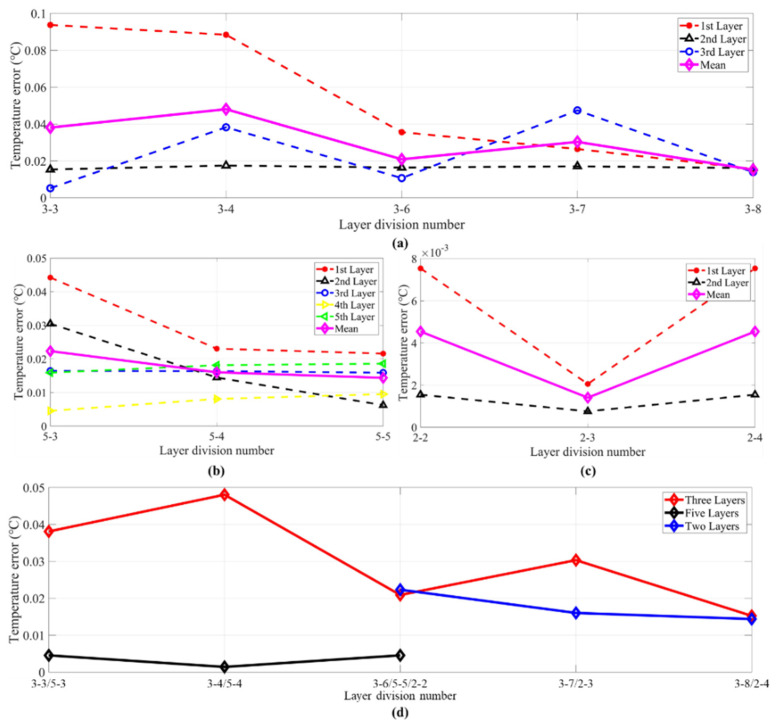
Inversion errors of the second group of **S1–S2**. The meanings of (**a**–**d**) are the same as in Figure 16a–d.

**Figure 19:**
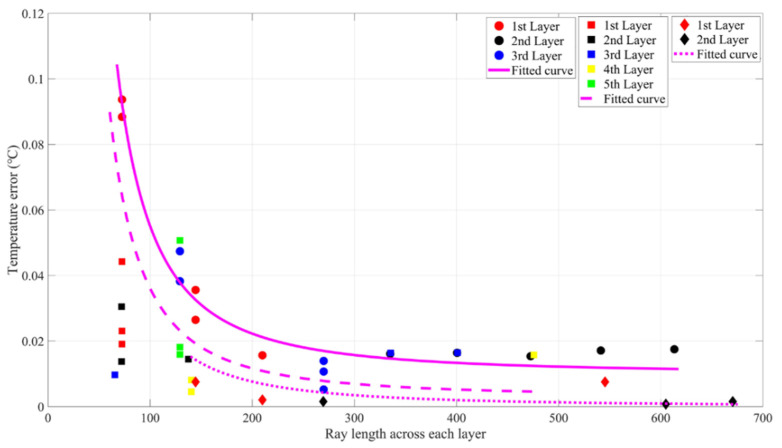
Relationship between the ray length across each layer and the inversion errors. The meanings of the labels are the same as in Figure 17.

**S1–S2** and **S2–S3** had the same grouping processing. The final results were similar, so only the second group could find appropriate λ to make the inversion error smaller than 0.05 °C. Figure 18 shows the inversion errors of the second group, and Figure 19 shows the relationship between the ray length across each layer and the inversion errors. From the results of **S1**–**S2**, a similar conclusion can be obtained.

(12) $$\mathrm{Three}\;\mathrm{layers}:\;{\hat{T}}_{err}=256.9*L^{-1.878}+0.011\;\mathrm{Five}\;\mathrm{layers}:\;{\hat{T}}_{err}=229.3*L^{-1.924}+0.003\;\mathrm{Two}\;\mathrm{layers}:\;{\hat{T}}_{err}=210.4*L^{-1.932}+0.001$$

Equation (12) is the equation of the three fitted curves in Figure 19. Using Equation (12) above, combined with Equation (11) (five layers were discarded), on a small scale, we can summarize that, there is an empirical relationship between the inversion error of every layer and the acoustic ray paths: ${\hat{T}}_{err}=a*L^{b}+c$. Where *a* can be equal to the direct diameter (D) minus the depth (d) a≈D−d. *B* was chosen to be around 1.8–2.0. *c* can be considered as a temperature error in the system. This relationship can be used for choosing a hierarchical approach in the data preprocessing. At the same time, it can be obtained from Figure 19 that different layers will improve the inversion error. However, the increase of layers will have a greater impact on the results, which needs to be considered.

In order to better summarize the conclusions, the meaningful speculations and conclusions in Section 3 are collected as follows:

(1) From the results of different settings of the same number of layers, the inversion error will reduce if the acoustic ray path (sound ray information) between different layers are of same length.

(2) If every layer has a whole ray path, the result has the smallest inversion error. Two layers containing the same ray path should be avoided. The layer that has the most ray paths passing through it will yield the smallest error and the most accurate results.

(3) Setting a preset value of temperature error can effectively improve the results, provided that λ has a solution.

(4) Different number of layers can improve the inversion error. However, when there are more layers, the results of every layer will be over-fitted, and when there are fewer layers, the results of every layer will have a larger difference. Therefore, the layering method needs to be selected reasonably.

(5) In a small-scale water body, the inversion error of every layer has a certain negative power relationship with the acoustic ray path length (sound ray information) obtained.

## 4. Conclusions

The stratification mechanism of reconstructing the water temperature field along vertical slices was analyzed in this study, and an empirical layer rule for vertical slices was also proposed. The layer-averaged water temperature and inversion error of each layer under different layer division types were studied. Finally, a layered error curve was obtained by analyzing the stratification division method and error of different layer division types. The inversion result will be of higher precision with these empirical layer rules.

The empirical layer rules obtained were as follows:

1. With a certain number of acoustic rays, each layer contains unique acoustic rays that are different from those in other layers; two layers that contain the same acoustic rays must be avoided. In short, every pair of two layers cannot contain only one information of a same acoustic ray at the same time.
2. After satisfying the first rule, the error of the layer-averaged analyzing method has a negative exponential relationship with the acoustic ray length of each layer. Therefore, each layer should include roughly the same ray length to reduce the inversion error.
3. The temperature inversion error can be decreased if the length of the acoustic rays contained in every layer is similar.
4. Setting a reasonable constraint value of temperature error and number of layers can improve the result. When the number of layers increase, the result may deviate.

By combining the water temperature analysis along the vertical slice, this article conducted preliminary research under certain acoustic rays with different layer widths. When preprocessing data, an empirical choice was provided to choose the layer method and better display the results. In the future, the analysis of multiple acoustic rays and multiple layers can be conducted to further summarize the error rules of the layer-averaged methods.

## Figures and Tables

**Table 1:** Parameters of the experimental setting.

Item	S1–S2	S2–S3
Central frequency	50 kHz	50 kHz
Transducer depth	20, 20 m	20, 16.9 m
Order of M sequence	10	10
Q ^1^ value	2	2
Station distance	270.07 m	224.04 m
Start and end time	15–16 September	15–16 September

^1^ Q value denotes the number of cycles per digit of M sequence.

**Table 2:** Five types of two layers.

Number	2–1	2–2	2–3	2–4	2–5
Length of 1st layer (m)	5	10	15	20	25
Length of 2nd layer (m)	25	20	15	10	5

**Table 3:** Ten types of three layers.

Number	3–1	3–2	3–3	3–4	3–5	3–6	3–7	3–8	3–9	3–10
Length of 1st layer (m)	5	5	5	5	10	10	10	15	15	20
Length of 2nd layer (m)	5	10	15	20	5	10	15	5	10	5
Length of 3rd layer (m)	20	15	10	5	15	10	5	10	5	5

**Table 4:** Five types of five layers.

Number	5–1	5–2	5–3	5–4	5–5
Length of 1st layer (m)	5	5	5	5	10
Length of 2nd layer (m)	5	5	5	10	5
Length of 3rd layer (m)	5	5	10	5	5
Length of 4th layer (m)	5	10	5	5	5
Length of 5th layer (m)	10	5	5	5	5

**Table 5:** Ray length and reference travel time (three rays and five layers).

S1–S2	Two Layers			Three Layers			Five Layers		
Ray Path	D	S	B	D	S	B	D	S	B
Layer 1	0	128.923	0	0	188.490	0	0	64.617	0
Layer 2	224.037	98.076	225.157	224.037	38.509	139.634	0	64.306	0
Layer 3	\	\	\	0	0	85.523	224.037	69.526	0
Layer 4	\	\	\	\	\	\	0	28.550	139.634
Layer 5	\	\	\	\	\	\	0	0	85.523
TL ^1^ (m)	224.037	226.999	225.157	224.037	226.999	225.157	224.037	226.999	225.157
TT ^2^ (s)	0.14962	0.15124	0.15061	0.14962	0.15124	0.15061	0.14962	0.15124	0.15061

^1^ TL denotes the travel length of acoustic ray paths.^2^ TT denotes the reference travel time of each ray.
